# Fabrication of Bio-Nanocomposite Based on HNT-Methionine for Controlled Release of Phenytoin

**DOI:** 10.3390/polym13152576

**Published:** 2021-08-03

**Authors:** Majid Abdouss, Nastaran Radgoudarzi, Alireza Mohebali, Elaheh Kowsari, Mojtaba Koosha, Tianduo Li

**Affiliations:** 1 Shandong Provincial Key Laboratory of Molecular Engineering, School of Chemistry and Chemical Engineering, Qilu University of Technology (Shandong Academy of Sciences), Jinan 250353, China; m_koosha@sbu.ac.ir; 2 Pharmaceutical Sciences Research Center, Department of Chemistry, Amirkabir University of Technology, No. 350, Hafez Ave, Valiasr Square, Tehran 1591634311, Iran; nastaran.radgoodarzi10@gmail.com (N.R.); arma@aut.ac.ir (A.M.); elahehkowsari@gmail.com (E.K.)

**Keywords:** HNT, wound healing, phenytoin, bio-nanocomposite

## Abstract

In this study, a novel promising approach for the fabrication of Halloysite nanotube (HNT) nanocomposites, based on the amino acid named Methionine (Met), was investigated. For this purpose, Met layered on the outer silane functionalized surface of HNT for controlled release of Phenytoin sodium (PHT). The resulting nanocomposite (MNT-g-Met) was characterized by FTIR, XRD, Zeta potential, TGA, TEM and FE-SEM. The FT-IR results showed APTES and Met peaks, which proved the modification of the HNTs. The zeta-potential results showed the interaction between APTES (+53.30) and Met (+38.80) on the HNTs (−30.92). The FE-SEM micrographs have displayed the grafting of Met on the modified HNTs due to the nanotube conversion to a rough and indistinguishable form. The amount of encapsulation efficiency (EE) and loading efficiency (LE) of MNT-g-Met was 74.48% and 37.24%, while pure HNT was 57.5%, and 28.75%, respectively. In-vitro studies showed that HNT had a burst release (70% in 6 h) in phosphate buffer while MNT-g-Met has more controlled release profile (30.05 in 6 h) and it was found to be fitted with the Korsmeyer-Peppas model. Due to the loading efficiency and controlled release profile, the nanocomposite promote a good potential for drug delivery of PHT.

## 1. Introduction

Chronic wounds are among the most costly unresolved health issues that reduce quality of life, costs, and acute conditions [1,2]. The wound healing process includes a sequence of events, including inflammatory responses, regeneration of the epidermis, shrinkage of the wound, and finally, connective tissue formation and remodeling. Timely and appropriate treatment using a drug delivery system can prevent wound infection development and its transformation into a chronic wound. Numerous drug delivery methods have been used to shorten the wound healing process and prevent infection, but there is still a long way to go [3,4].

One of the essential drugs that recently showed improvement in wound healing is Phenytoin sodium (PHT), which has been prescribed to treat epilepsy since 1973 [5]. A common side effect of PHT was the overgrowth of gingival fiber cells, which affected the formation of connective tissue cells, leading to its use in wound healing [6,7]. The topical application of PHT increases extracellular material, and connective tissue protein decreases collagenase enzyme and increases collagen production, which in turn, accelerates granulation tissue and accelerates wound healing. It also increases the division of fibroblasts by increasing estrogen, thus, accelerating healing. Many studies have shown the topical effect of PHT on wounds [8,9,10,11,12]. PHT release has been studied from various polymeric carriers, prepared via synthetic approaches, such as electrospinning [13]. 

Halloysite nanotube (HNT) is a mineral nanoclay that can be a suitable PHT carrier due to its remarkable properties. Recent studies have implemented HNTs for the controlled release of topical and gastrointestinal drugs due to their biocompatibility, high mechanical strength, low cost, hollow and nano-sized tubular structure [14,15,16,17,18,19,20,21]. HNT is a multilayer aluminosilicate (${\mathrm{Al}}_{2}{\mathrm{Si}}_{2}O_{5}{\left(\mathrm{OH}\right)}_{4}.nH_{2}$*O* [22]. While, the outer layer structure consists of silanol groups (Si–OH). Its inner surface comprises Al-OH groups, which causes differences in electrical charge on the outer and inner surfaces [23,24,25]. Therefore, at a pH between 2 and 8, the outer layer has a negative electric charge, and the inner layer (lumen) has a positive electric charge. Morphologically, the inner diameter of the HNT lumen is 10–15 nm, and the outer diameter is about 50–100 nm, which prepares it to be a useful carrier of drugs of suitable size [17,26,27,28,29]. Although the use of HNT as a suitable carrier in drug delivery can be beneficial, and the release of the drug from its lumen surface shows a specific burst effect that is not suitable for controlled release. To prevent this phenomenon and control the drug release from HNT, modification of its external surface by silane group or some polymers can be useful [28,30,31]. 

One of the agents that can be interesting in modifying of HNT outer surface is amino acids, which in addition to being biocompatible, are very effective in improving drug release [32,33,34]. Surface modifying the HNT with amino acids can control the drug’s release and effectively reduce its side effects. The layering of HNT surface by amino acid, as a novel approach, can be carried out by creating a covalent bond with the outer surface of HNT by a silane agent such as APTES [35,36,37]. Methionine (Met) is an α-amino acid used in the biosynthesis of proteins. An α-carboxylic acid group and an S-methylthio ether side chain classify it as a non-polar aliphatic amino acid. In the process of layering on HNT, the acidic agent of Met binds to the NH_2_ group of APTES molecule, which grafted on the surface of HNT, and the resulting nanocomposite will have a positive effect on drug loading and release of PHT. The use of amino acids, such as Met, is a novel approach to modifying HNT surfaces [38]. 

In this study, MNT*-g-*Met nanocomposite preparation as a PHT carrier for chronic wound healing was investigated. First, the HNT outer surface was modified by APTES, and then Met was grafted on it. Fourier-transform infrared spectroscopy (FT-IR), Zeta potential, scanning electron microscope (SEM), transmission electron microscopy (TEM), thermogravimetric analysis (TGA), differential scanning calorimetry (DSC), X-ray diffraction analysis (XRD), and drug loading tests were used to characterize the prepared nanocomposite. The in-vitro drug release of the nanocomposite was performed via the dialysis bag method. PHT release kinetics were investigated based on four mathematical models: Zero-order, first-order, Higuchi, and Korsmeyer-Peppas. The data obtained from the mentioned models were processed and evaluated based on the correlation coefficient R^2^.

## 2. Materials and Methods

### 2.1. Material

HNT (Halloysite nanoclay, Latah County, 30–70 nm × 1–3 μm, nanotube), (3-aminopropyl) triethoxysilane (APTES), L-Methionine (Met, reagent grade, ≥98% for HPLC), triethanolamine (TEA), sulfuric acid, toluene, and dimethylformamide (DMF) were purchased from Sigma Aldrich. PHT (Phenytoin, Pharmaceutical Secondary Standard, purity > 99.9%) was received from Safeway Research Center (S.R.C.). All chemicals used as received. The distilled water was used throughout the experiments.

### 2.2. HNT Purification

To remove HNT impurities, it must be purified. Briefly, to have an ideal dispersion of HNT in water, a drop of polysorbate-80 (a nonionic surfactant) was first added to 50 mL of deionized water at 60 °C. HNT (500 mg) was added to the solution while on a magnetic stirrer at 60 °C and 1000 rpm for 18 h. Then washed and centrifuged three times with distilled water, and the remaining residue was dried at 100 °C for 10 h [14,39]. 

### 2.3. HNT Modification with APTES

The functionalization of HNT with APTES is one of the most widely used methods for linking polymers or other active functions on HNT outer and inner surfaces. As mentioned in previous articles [14], 1 g HNT is added to 20 mL of toluene, sonicated, and placed on a magnetic stirrer at 600 rpm for 1 h. Then, 1 mL of TEA and APTES were added dropwise to the resulting suspension while stirring. The resulting suspension was refluxed under the nitrogen atmosphere for 16 h. The HNT@APTES entitled MNT (Modified HNT) was washed three times with water and ethanol and dried in a vacuum oven (~1 bar) at 40 °C overnight [40,41,42,43].

### 2.4. Preparation of HNT-g-Met Nanocomposites

**Figure 1:**
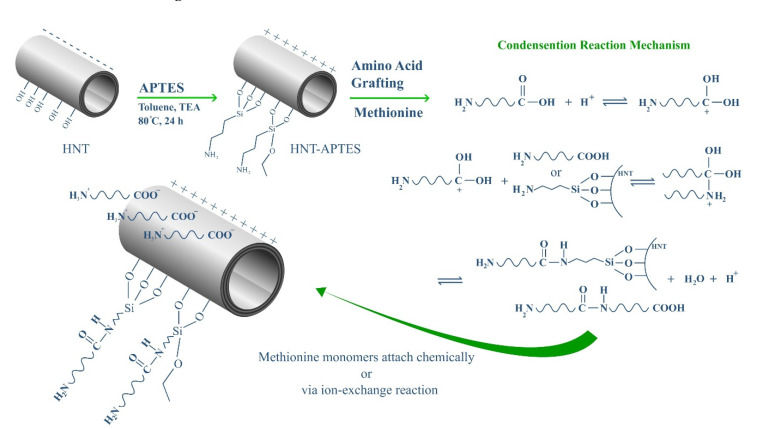
Schematic of surface modification of HNT and facial fabrication of MNT*-g-*Met.

According to Figure 1, to create suitable functional groups for amino acid uptake, first, APTES was placed on the HNT surface. APTES has an amine group that can bind to Met’s carboxyl group to form the final nanocomposite. For this purpose, 200 mg MNT was dispersed in 20 mL of DMF, and a few drops of 0.1 M sulfuric acid were added to bring the pH of the mixture to 4. Then, 1 mmol of Met was added to the resulting suspension and refluxed at 80 °C for 24 h. The final nanocomposite (HNT*-g-*Met) was washed three times with distilled water and ethanol and dried in a vacuum oven (~1 bar) at 50 °C overnight [44].

### 2.5. PHT Loading Studies

A vacuum technique was used to load the PHT into the HNT lumen. This technique is based on the difference between the vapor pressure of the solution in the lumen and the bulk solution, which pushes the drug solution into the lumen and subsequently provides hydrogen and electrostatic bonds between PHT’s negative charge, the positive charge of the inner surface of HNT. In this method, the nanocomposite was dried, then 100 mg of the HNT*-g-*Met or MNT were added to the PHT solution (1 mg/mL) separately. The mixture was sonicated for 30 min. It was then placed in a vacuum jar (0.08 bar) for 20 min and then exposed to atmospheric pressure for 10 min after the vacuum breaks down. This process was repeated three times to achieve more load efficiency. The amount of drug-loading was measured by HPLC chromatography at 220 nm. The encapsulation efficiency (EE) and loading efficiency (LE) were calculated using the amount of free PHT by Equations (1) and (2) [45,46,47]:

(1) $$EE\left(\%\right)=\frac{\left(total\;amount\;of\;PHT-free\;PHT\right)}{\left(total\;amount\;of\;PHT\right)}\times 100$$

(2) $$LE\left(\%\right)=\frac{\left(weight\;of\;loaded\;PHT\right)}{\left(total\;weight\;of\;nanocomposite\;and\;loaded\;PHT\right)}\times 100$$

### 2.6. In Vitro Drug Release Studies

The in-vitro dissolution test was performed using a dialysis bag based on the PBS diffusion phenomenon (pH = 7.4) at 32.5 °C [48]. In this method, 5 mg of the loaded nanocomposite was dispersed in PBS solution in a dialysis bag and placed in 30 mL of release medium and placed at 32.5 °C on the stirrer [49]. At specific times, 2 mL of the release solution was removed, and the amount of released drug was determined by HPLC at 220 nm [50]. 

### 2.7. Characterization

FT-IR was used to study the functional groups and possible interactions in the prepared samples. For this purpose, samples were pressed under 1.3 × 10^–5^ bar (0.01 torr) with KBr and were examined in the range of 400 to 4000 cm^−1^. Zeta-potential results of the prepared sample in ethanol at pH = 7.4 using the SZ-100z dynamic light scattering and voltage range of ±200 mV were used to investigate particle surface charge changes and the stability of colloidal dispersions. The thermal degradation behavior of samples was studied using thermogravimetric analysis (TGA) from room temperature to 800 °C with a rate of 20 °C/min in the nitrogen atmosphere. The particle’s appearance and morphology were studied using scanning electron microscopy (SEM), the TESCAN MIRA3 LMU model and transmission electron microscopy (TEM) test, using a ZEISS EM10C model with a voltage of 80 kW. The X-ray diffraction (XRD) is a rapid analytical technique to determine the samples’ crystal structure, which was performed using the Siemens D5000 (Germany) instrument at 25 °C.

### 2.8. Statistical Analysis

The statistical analysis for the determination of reproducibility in the loading, zeta potential, and release test was performed using one-way ANOVA statistical analysis. The obtained data were considered to be significantly different at *p* < 0.05. All data are presented as mean values with standard error (mean ± SD), n = 3 in each test.

## 3. Results and Discussion

In this study, HNT was used to prepare a new nanocarrier for wound dressing using PHT. HNT was considered for loading capacity, biocompatibility, and tubular structure. For the first time, a new approach was used to prepare HNT-based nanocomposites using amino acids grafted on the surface of HNT. In the following, the effect of Met on drug release was investigated.

### 3.1. Physicochemical Studies

**Figure 2:**
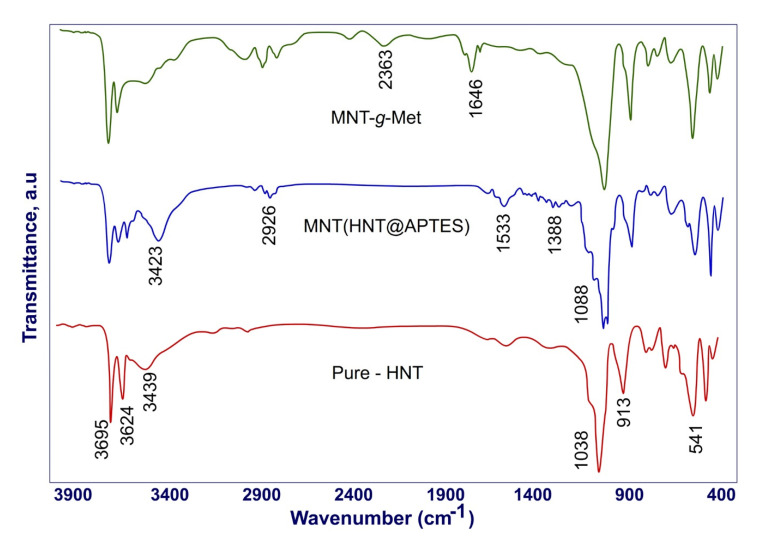
FT-IR spectra of HNT, MNT and, MNT*-g-*Met.

Due to covalent bonds forming during the nanocomposite preparation process, it is necessary to study the bond strengths and compare them. For this purpose, the FT-IR spectrum of HNT, MNT, and MNT-*g*-Met are shown in Figure 2. In this figure, the HNT spectra before and after surface modification are marked. In the pure HNT spectrum, the peaks appearing at 3624 cm^−1^ and 3695 cm^−1^ are related to the inner, and interlayer hydroxyl groups’ vibration, respectively. The peak shown in 913 cm^−1^ indicates the vibration of Al_2_OH. Symmetric stretching vibration of Si–O–Si appeared at 1038 cm^−1^ [47]. After surface modification of HNT with APTES, the new peak appears in 3423 cm^−1^ related to the amine groups in APTES. The two peaks in 2926 cm^−1^ and 1388 cm^−1^ are related to the CH_2_ group, respectively. The C-N peak appeared in 1088 cm^−1^. Another peak shown at 1533 cm^−1^ in the HNT-APTES spectrum belongs to the NH_2_ group. After the Met graft on the HNT surface, new peaks appeared that indicated a covalent bond between Met and APTES. The peak shown in 2363 cm^−1^ corresponds to S–CH_3_ of Met. Another short peak at 1646 cm^−1^ indicates the carbonyl group in the nanocomposite structure. 

**Figure 3:**
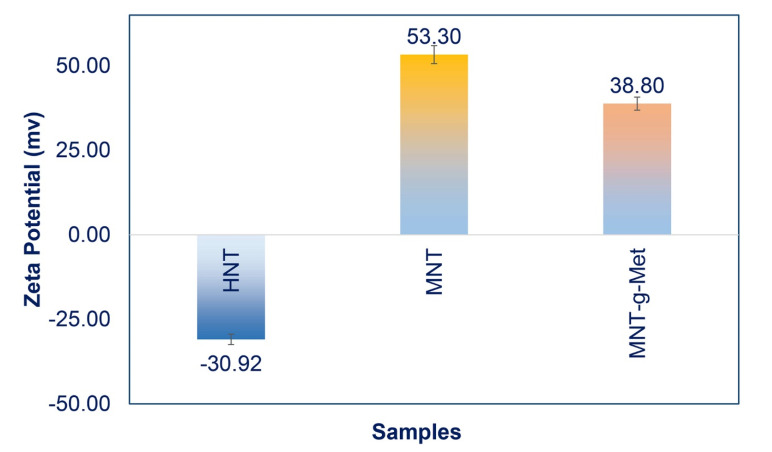
Zeta potential of HNT, MNT and, MNT*-g-*Met.

The surface modification of HNT was successfully proved by changing the surface charge during the process. Figure 3 shows a comparison of pure HNT surface charge, MNT, and HNT*-g-*Met. Pure HNT has a negative zeta potential of −30.92 mV. After modifying of HNT surface with APTES, its negative charge was naturalized to +53.3 mV. By grafting Met on MNT, the amount of zeta potential was changed to +38.8 mV, which is related to the NH_3_^+^ group of amino acid layering on the surface of the HNT. These results can prove that HNT was chemically layered with a positively charged amino acid.

**Figure 4:**
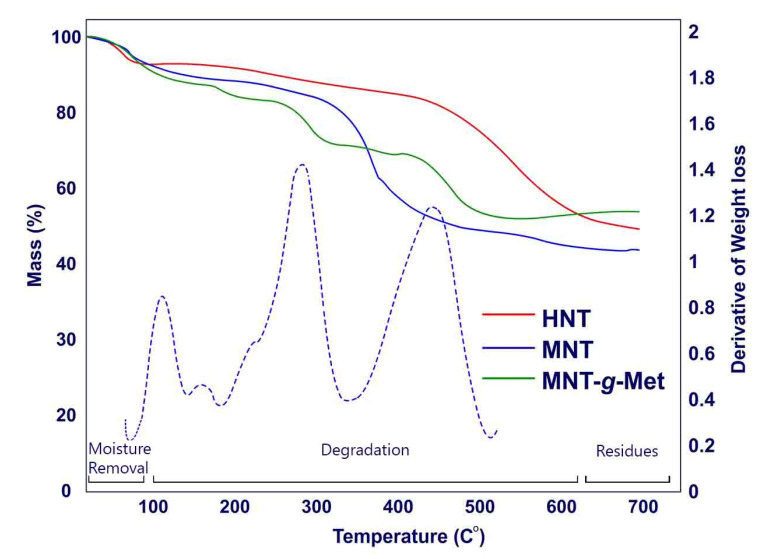
Tg analysis of HNT (solid red line), MNT (solid blue line), MNT-g-Met (solid green lines), and DTG diagram of MNT-g-Met (blue dotted line).

Figure 4 presented TG analysis of prepared samples. The results obtained from the HNT curve show two events. The first event is about a 20% mass reduction that occurred at 50–100 °C. At this stage, the trapped water inside the HNT layers evaporates. The second event is related to the dehydroxylation of the Al-OH group in the HNT structure. This phenomenon at 500 °C leads to 21% weight loss. The TGA curve of MNT shows a higher weight loss than HNT between 250 °C and 400 °C, which is related to the decomposition of APTES. As shown in this figure, the fabricated HNT*-g-*Met nanocomposite decomposes in several thermal events [43]. The first event is related to the evaporation of water from its structure, which occurs at about 100 °C. Following this event, Met was decomposed at 250 °C, reducing the nanocomposite weight by about 12%. At 450 °C, HNT was dehydroxylated with a 10% weight loss. Figure 4 also shows the MNT*-g-*Met DTG diagram for better differentiation of the TGA diagram, which confirms the above studies. 

**Figure 5:**
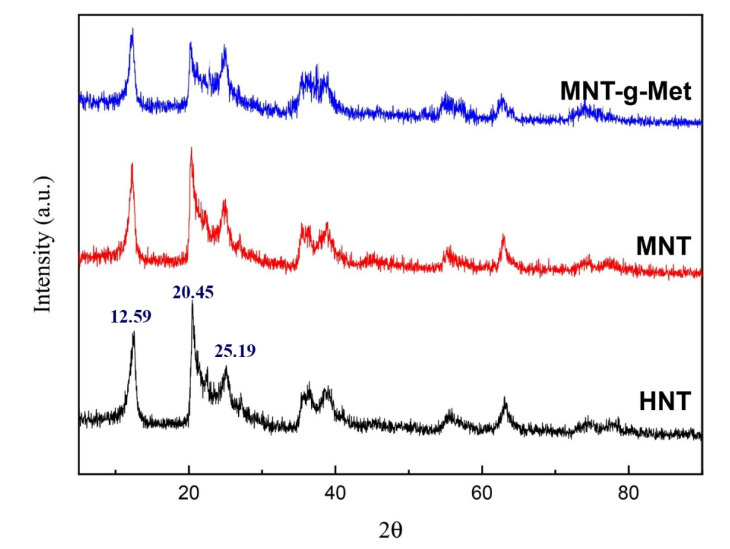
X-ray diffraction diffractograms of HNT, MNT and, MNT-g-Met.

The XRD pattern of HNTs, MNT, and MNT*-g-*Met presents in Figure 5. The XRD diagram of pure HNT shows the basal reflection at 2Ɵ = 12.59°, which is related to the HNT crystalline structure’s multilayer wall. According to Bragg’s law, the distance between plates is 7.5Å. The two peaks at 2Ɵ = 20.45° and 2Ɵ = 25.19° indexed to the plates of 110 and 002, respectively [51]. Also, no peak appears in 2Ɵ = 8.1° to be related to HNT, indicating that HNT is water-free [47]. Figure 5 also shows two diffractograms for MNT and MNT*-g-*Met. After modification of HNT by APTES (MNT), the reflection of pure HNT remained unchanged, indicating that the APTES surface grafting was not affected the basal spacing and crystalline structure. This result confirms that APTES was not inserted into the space between the HNT layers. As shown in the diffractogram of MNT*-g-*Met, since Met does not have a specific crystalline structure, Met grafting does not affect MNT structure.

### 3.2. Morphology Studies

**Figure 6:**
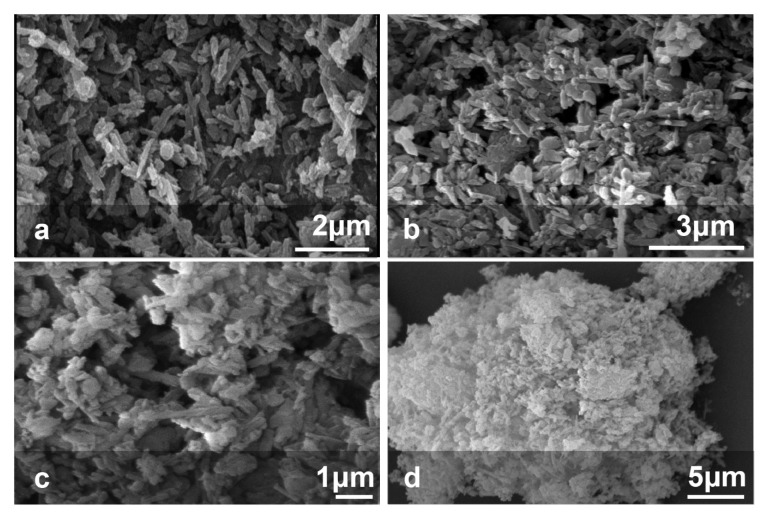
SEM micrograph image of pure HNT (**a**), MNT (**b**), MNT-*g*-Met (**c**) and, (**d**) MNT-*g*-Met.

Figure 6 shows the SEM images of HNT, MNT, and MNT*-g-*Met nanocomposite. As shown in Figure 6a, pure HNT has a neat tubular structure. After modifying the HNT surface with APTES (Figure 6b), its morphology is preserved and does not change significantly. As the Met grafts on the outer surface, it becomes slightly rougher and more extensive, as shown in Figure 6c,d. Although, a slight accumulation is observed in the nanocomposite structure, during the surface modification process, the HNT tubular structure remains unchanged.

**Figure 7:**
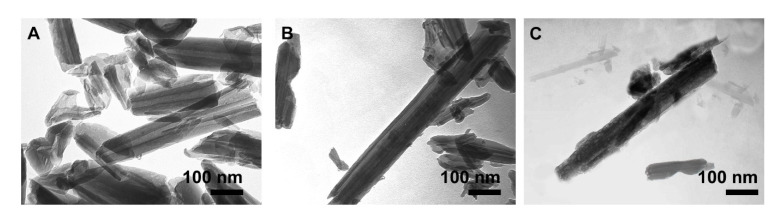
TEM images of the pure HNT (**A**), MNT, (**B**) and MNT-*g*-Met (**C**).

According to the TEM images, the tubular morphology of pure-HNT with hallow lumen was observed in Figure 7. The lumen diameter of HNT is about 17–20 nm and length average is 400 nm with a smooth surface, and completely open (Figure 7A). After modifying the outer surface of the HNT with APTES (MNT), the outer wall becomes slightly thicker, making the lumen less recognizable (Figure 7B). Figure 7C shows that the Met was grafted onto HNT surface and a heterogeneous, high porous structure was obtained. As can be seen in the Figure 7A–C, the HNT tubular structure is preserved throughout the modification process.

### 3.3. Loading Studies of PHT

The LE and EE of different samples were evaluated in PBS 7.4. Loading was performed based on the vacuum technique. The amount of drug-loaded was calculated based on the difference between the initial drug concentration and the free drug in the bulk solution. Table 1 shows the LE and EE for pure HNT, MNT, and MNT*-g-*Met. The results show that when functionalized HNT was grafted with Met, the PHT loading was increased. This was due to the positive charge of Met and APTES and the electrostatic attraction between the negatively charged PHT and the amine group of these molecules. Due to the PHT molecule structure, hydrogen bonds can also increase LE and EE, although this increase is not significant with the Met layering.

### 3.4. In Vitro Drug Release Studies

**Figure 8:**
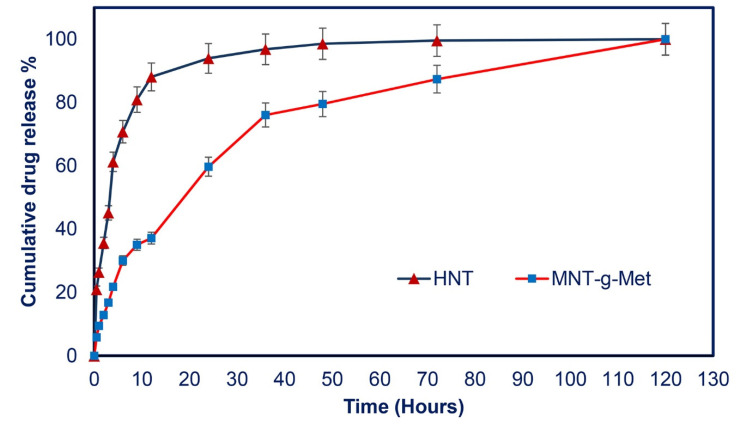
The in-vitro drug release profile of HNT and MNT-*g*-Met.

Figure 8 shows PHT’s cumulative release profile from loaded HNT and MNT*-g-*Met nanocomposite in PBS at pH 7.4. According to the diagram, the cumulative release of PHT from pure HNT reached 70.7% after 6 h, related to the burst effect. This phenomenon results in a lack of controlled drug release, which is not desirable for a drug delivery system. After 48 h, the cumulative release of PHT reached an almost constant concentration. The burst effect in the initial stage is due to the dissolution of drug molecules adsorbed on the outer surface and layer of the HNT. In the next step, PHT release from the HNT lumen was slower due to the strong absorption of drug molecules. It is expected that this burst release problem will be partially remedied by modifying the HNT surfaces with Met. The PHT release profile from MNT*-g-*Met progressed more rapidly in the early hours (after 6 h: 30.05%) of release, but no burst effect was observed. This rapid release was due to drugs adsorbed on the Met surface. This rapid release was due to drugs adsorbed on the Met surface. When Met is grafted onto the outer surface of functionalized HNT (MNT), the amino acid molecule blocks the HNT lumen’s output to such an extent that it leads to a controlled and sustained release of the PHT. The results show that the fabricated nanocomposite can act as a carrier for drug delivery to chronic wounds. 

**Figure 9:**
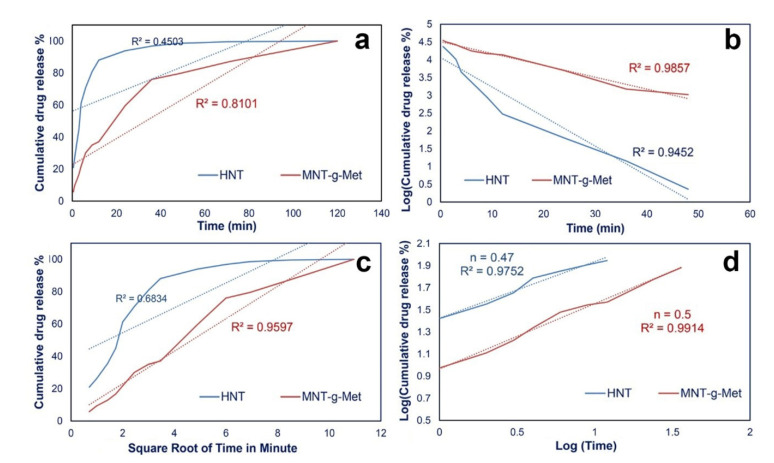
Drug release data fitted to kinetic models: Zero-order (**a**), first order (**b**), Higuchi (**c**) and, Korsmeyer-Peppas (**d**).

The release kinetics of PHT from MNT*-g-*Met are presented in Figure 9. The release data were fitted with zero order, first order, Higuchi and, Korsmeyer-Pappas equation models. The correlation coefficient was calculated in each model. The results showed that the release kinetic pf PHT from MNT*-g-*Met in PBS fitted by Korsmeyer-Peppas equation, which is expressed as follows [52]:

(3) $$\frac{Mt}{M\infty }=kt^{n}.$$

*Mt/M∞* is the fractional amount of PHT that release at time *t*, *k* is a constant value, and n is the release exponent. 

Table 2 shows these kinetic parameters for PHT release from pure HNT and MNT*-g-*Met. According to the obtained results, the n values for HNT and nanocomposite were about 0.47 and 0.5, respectively. An *n* value less than 0.5 indicates that the release follows a Fickian diffusion from polydisperse systems. The results also show that the nanocomposite particles possess a smaller k value than the pure HNT, which indicates a controlled and sustained release in the same condition [53].

## 4. Conclusions

In this study, a novel approach for the preparation of HNT nanocomposites, based on the amino acid graft on the outer surface of HNT, was proposed. For this purpose, HNTs and Met were selected as the encapsulating agents for improving the PHT loading and release, and APTES was selected as a linker for making a chemical bond between the HNTs and Met. The MNT*-g-*Met nanocomposite was fabricated via functionalizing HNT with APTES, followed by Met grafting on its outer surfaces. The prepared nanocomposite was presented as a suitable carrier for PHT for use in wound dressing. The nanocomposite’s physicochemical properties were studied by FT-IR, FESEM, TGA, XRD, and zeta potential, which all confirmed the success of the composite preparation. The morphological results presented the structure of uniform nanotube particles. The results indicated that the prepared nanocomposite of MNT-g-Met could improve the amount of PHT from 28.75% to 52.61 wt% compared with the pure HNTs. In-vitro studies showed that PHT release from HNT occurs very rapidly (k = 26.57/h), while this process is controlled in MNT-g-Met (k = 9.22/h). The Korsmeyer-Peppas model fitted the release kinetics, which indicated the Fickian diffusion mechanism from cylindrical multilayer structure. Finally, all the results show that the prepared nanocomposite can be a suitable carrier for PHT or similar drugs to heal chronic wounds.

## Figures and Tables

**Table 1:** Drug Loading Efficiency (LE) and Encapsulation Efficiency (EE) of the prepared sample.

Sample Name	EE%	LE%
HNT	57.5 ± 0.04	28.75 ± 0.23
MNT	43.45 ± 0.07	39.20 ± 0.20
MNT*-g-*Met	51.22 ± 0.14	52.61 ± 0.15

Each value demonstrate the mean ± SD (n = 3).

**Table 2:** Kinetic parameters for PHT release.

Sample Name	K/h	n	R^2^
HNT	26.57	0.47	0.9752
HNT*-g-*Met	9.22	0.5	0.9914

## Data Availability

The data presented in this study are available on request from the corresponding author.
